# Molecular Insights into the Pathogenesis of Alzheimer's Disease and Its Relationship to Normal Aging

**DOI:** 10.1371/journal.pone.0029610

**Published:** 2011-12-28

**Authors:** Alexei A. Podtelezhnikov, Keith Q. Tanis, Michael Nebozhyn, William J. Ray, David J. Stone, Andrey P. Loboda

**Affiliations:** 1 Exploratory and Translational Sciences, Merck Research Laboratories, Merck Inc., West Point, Pennsylvania, United States of America; 2 Neuroscience, Merck Research Laboratories, Merck Inc., West Point, Pennsylvania, United States of America; Biological Research Center of the Hungarian Academy of Sciences, Hungary

## Abstract

Alzheimer's disease (AD) is a complex neurodegenerative disorder that diverges from the process of normal brain aging by unknown mechanisms. We analyzed the global structure of age- and disease-dependent gene expression patterns in three regions from more than 600 brains. Gene expression variation could be almost completely explained by four transcriptional biomarkers that we named BioAge (biological age), Alz (Alzheimer), Inflame (inflammation), and NdStress (neurodegenerative stress). BioAge captures the first principal component of variation and includes genes statistically associated with neuronal loss, glial activation, and lipid metabolism. Normally BioAge increases with chronological age, but in AD it is prematurely expressed as if some of the subjects were 140 years old. A component of BioAge, Lipa, contains the AD risk factor *APOE* and reflects an apparent early disturbance in lipid metabolism. The rate of biological aging in AD patients, which cannot be explained by BioAge, is associated instead with NdStress, which includes genes related to protein folding and metabolism. Inflame, comprised of inflammatory cytokines and microglial genes, is broadly activated and appears early in the disease process. In contrast, the disease-specific biomarker Alz was selectively present only in the affected areas of the AD brain, appears later in pathogenesis, and is enriched in genes associated with the signaling and cell adhesion changes during the epithelial to mesenchymal (EMT) transition. Together these biomarkers provide detailed description of the aging process and its contribution to Alzheimer's disease progression.

## Introduction

During normal aging the brain undergoes many changes resulting in a *gradual* but detectable cognitive decline that is associated with limited neuronal loss, glial proliferation in the cortex, and gross weight decrease of 2–3% per decade [1], [2]. On the molecular level, the mechanisms driving aging of the brain are not yet understood, but likely include mitochondrial DNA damage [3] and chronic oxidative stress [4]. This slow decline in cognitive ability does not interfere with normal function through at least 100 years of life. In contrast, Alzheimer's disease (AD) is a debilitating neurodegenerative disorder associated with a *rapid* cognitive decline with an average survival of 5–10 years after the diagnosis [5], [6], [7]. Age is the main AD risk factor with almost half of the population over age 85 affected. AD, however, clearly differs from the normal aging in that it causes dramatic loss of synapses, neurons and brain activity in specific anatomical regions, and results in massive atrophy and gliosis [1], [8].

The factors that cause some individuals to depart from the relatively benign process of normal brain aging and instead undergo the pathological cascade that leads to AD are unknown. A number of genetic risk factors for AD have been proposed [9], [10], [11], [12], however only the apolipoprotein E (*APOE*) ε4-allele, which lowers the age of onset and accelerates the cognitive decline, has a large effect [13], [14]. Pathologically AD is characterized by the presence of two insoluble protein aggregates, senile plaques formed from the peptide β-amyloid (Aβ) and neurofibrillary tangles composed of hyperphosphorylated tau protein [15]. In rare familial AD, the cause of disease is autosomal dominant mutations in Aβ precursor protein (*APP*) or the Aβ-producing enzymes presenilins (*PSEN1* or *PSEN2*), which are all thought to lead to increased levels of aggregated Aβ [9], [10], [16]. Likewise, mutations in tau (*MAPT*) that predispose it to aggregation can cause specific diseases that involve profound neurodegeneration and dementia [17], [18]. Thus, like in other neurodegenerative diseases such as Huntington's disease (HD) and Parkinson's disease, the formation of toxic insoluble aggregates seems to be a key pathogenic step. However, it is not known why these Aβ and tau aggregates accumulate in AD patients nor how they contribute to neuronal dysfunction, particularly for Aβ deposits, which can often be found in the brains of elderly non-demented subjects [19].

An important goal of AD research is to identify interventions that maintain brain function, potentially by inhibiting the formation or improving the clearance of neurotoxic aggregates, or by promoting resistance to, or recovery from, damage. A number of biological processes have been associated with AD, including cholesterol metabolism, inflammation, and response to misfolded proteins such as increased expression of heat shock proteins [20]. The link with lipid metabolism is supported for example by the essential role of APOE in lipid transport in the brain [13], [14]. However, these processes have not been unequivocally ordered into a pathogenic cascade, and the molecular mediators and correlates of each are largely unknown. Microarray gene expression profiling provides an opportunity to observe processes that are common for normal aging, AD, and other neurodegenerative diseases, as well as to detect the differences between these conditions and disentangle their relationships. We profiled over six hundred postmortem samples assembled in the Harvard Brain Tissue Resource Center (HBTRC, McLean Hospital, Belmont, MA). We used metagene (factor) analysis [21], [22], [23], [24], [25] to distinguish several major gene expression patterns involved in brain aging and disease and to quantitatively define the corresponding biomarker scores. The correlation analysis of the biomarker scores between three profiled brain regions revealed systemic effects of the same disease processes on different brain regions. We also propose a model of Alzheimer's disease progression that specifies the complex sequence of molecular pathological events associated with the disease that are driven by aging, which appears as the main factor in the disease initiation and progression [1], [8].

## Methods

### Study Population and Sample Collection

The dataset comprises gene expression data from brain tissues that were posthumously collected from more than 600 individuals with AD diagnosis, HD diagnosis, or with normal non-demented brains. All brains were obtained from individuals for whom both the donor and the next of kin had completed the Harvard Brain Tissue Resource Center Informed Consent (form available at: http://www.brainbank.mclean.org/PDF files/Consent.pdf). All tissue and research were conducted according to the Harvard Brain Tissue Resource Center Guidelines (including: HUMAN TISSUE HANDLING RISKS & SAFETY PRECAUTIONS agreement; HUMAN TISSUE SINGLE USER agreement, and HBTRC ACKNOWLEDGMENT agreement, available at http://www.brainbank.mclean.org/PDF files/TissueRequest.PDF). The study was approved by McLean Hospital Institutional Review Board.

Braak stage [26] and atrophy were assessed by pathologists at McLean Hospital (Belmont, MA). Only neuropathologically confirmed AD subjects with Braak >3 were included in this profiling experiment. The profiled brain regions included dorsolateral prefrontal cortex (PFC, Brodmann area 9), visual cortex (VC, Brodmann area 17), and cerebellum (CR). These regions were chosen because PFC is impacted by the AD pathology while the latter two regions remain largely intact throughout most of the disease [26]. The tissue samples from subjects with HD diagnosis, while not the main focus of this work, were included to compare the two neurodegenerative diseases. The samples were flash frozen in liquid nitrogen vapor with an average postmortem interval (PMI) of about 18 hours. The clinical and demographic information for this study, including diagnosis, gender, age at the time of death, PMI, and Braak stage, is summarized in Table S1.

### Gene Expression Profiling

A total of 1 µg of mRNA extracted from each tissue sample was amplified to fluorescently labeled cRNA, and profiled by the Rosetta Gene Expression Laboratory in two phases using the Rosetta/Merck 44k 1.1 microarray (GPL4372) (Agilent Technologies, Santa Clara, CA) [27]. The average RNA integrity number of 6.81 was sufficiently high for the microarray experiment monitoring 40,638 transcripts representing more than 31,000 unique genes. The expression levels were processed and normalized to the average of all samples in the batch from the same region using Rosetta Resolver (Rosetta Biosoftware, Seattle, WA).

We refer to each batch of tissue samples using the abbreviated brain region and the phase of the experiment (e.g., PFC2 refers to prefrontal cortex samples profiled in phase 2). Table S1 summarizes the number of samples in each category. All microarray data generated in this study are available through the National Brain Databank at the Harvard Brain Tissue Resource Center (http://national_databank.mclean.harvard.edu/brainbank/Main). Any researcher wishing to obtain the de-identified dataset can do so by contacting the National Brain Databank at McLean Hospital, Harvard Medical School.

This microarray dataset is MIAME compliant. The raw and final processed data for each hybridization are available to any researcher upon request. The essential sample annotation including experimental factors and their values (e.g., gender, age, PMI, pH) is available and summarized in Table S1. The study utilized a standard annotated microarray (GPL4372) and standard pipeline of data processing for this array.

### Data Analysis

We used the log10-ratio of the individual microarray intensities to the average intensities of all samples from the same brain region profiled in the same phase as the primary measure of gene expression. Quality control of gene expression data was performed by principal component analysis using MATLAB R2007a (Mathworks Inc., Natick, MA). Outlier samples (less than 2%) were removed from the data set based on extreme standardized values of the first, second, or third principal components, with absolute *z*-scores more than 3.

The first principal component (PC1) was used to assess the major pattern of gene expression variability in the dataset. Genes that were highly correlated with PC1 were used to build a surrogate biomarker. Throughout this work we used Pearson correlation coefficients, ρ, and assessed their significance, *p*, assuming normal distribution for Fisher z-transformed values, atanh ρ [28]. Significant differential expression for each gene was evaluated using t-test p-values [28]. Multiple-testing correction of p-values was done according to Benjamini-Hochberg procedure to obtain false-discovery rates (FDR) [29]. These analyses were performed using Statistical Toolbox of MATLAB R2007a (Mathworks Inc. Natick, MA).

Gene expression changes associated with aging and disease were characterized by metagenes combining sets of genes with significant association with a disease trait and a very strong Pearson correlation with each other. We utilized a procedure of exploring covariance structure of the gene expression data similar to metagene identification [22], factor analysis of gene expression [23], and supervised gene module discovery [21], [24], [25]. Instead of genome-wide search for metagenes followed by analysis of associations between metagenes and disease traits, we used a supervised approach. After selecting genes significantly associated with the disease, we agglomeratively clustered them using Pearson correlation as a distance measure. Especially tight and large clusters in the dendrogram were then assigned to metagenes, i.e., the dendrogram was cut so that several hundred genes in a branch qualified for a metagene and the average of their correlations to the mean (coherence) was not weaker than 0.75. We recognized that some metagenes could have two anti-correlated arms representing opposite trends in the gene expression (e.g., genes that are up- and downregulated with the end point).

The biological nature of the metagenes was assessed using the gene set overlap analysis with known biological processes described in GeneGo or Ingenuity. Significance of the overlaps between the lists was assessed using Bonferroni-corrected p-values [28], using Merck's proprietary Target and Gene Information system.

### Biomarker Scoring

Throughout this work we adopted the term *biomarker* to refer to a metagene together with associated score that quantifies it in each brain tissue sample. The biomarker score for each sample was calculated as the mean expression levels of the comprising gene probes or as the arithmetic difference between the means in the positive and negative arms of the metagene when both arms were specified.
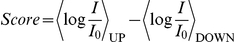
where *I*/*I*_0_ is normalized intensity of the metagene probes. To produce a robust score, all samples have to be normalized to the same reference. The reference intensity *I*_0_ for each gene corresponded to the average intensity in the cohort. Importantly, averaging genes that correlate with each other produced a measure that is more accurate than individual genes. For all metagenes identified in this work, the biomarker score represented a quantitative measure of a particular disease aspect in each brain sample. To evaluate the performance of the biomarker score as a classifier between diseased and normal samples, we used the area under the curve for the receiver operating characteristic (AUROC) [30].

### In Silico Experiments

To validate biomarkers identified in this work, we tested their coherence and predictive power in the context of independent gene expression dataset, GSE1572 [3]. This data set contained gene expression data from PFC samples of 30 non-demented subjects, aged 26–106. These samples were profiled on Affymetrix Human Genome U95 Version 2 Array (GPL8300). To select the probes and calculate the biomarker score, we matched the biomarker gene symbols to those represented on the HG-U95Av2 array. We calculated the correlation of the expression level of each selected probeset with the composite biomarker score (see above) and refined the selection by dropping probes that demonstrated opposite regulation according to the sign of the correlation coefficient. Finally, we correlated the biomarker scores with the subject age.

An additional set of public gene expression data used to validate the coherence and predictive power of the biomarkers was obtained from hippocampus samples from elderly control and AD subjects, GSE1297 [31], [32]. These 31 samples were profiled using Affymetrix Human Genome U133A Array (HG-U133A). To select the probes and calculate the biomarker score, we matched the biomarker gene symbols to those represented on the array, refined them as described above, and averaged the gene expression values according to the equation in the previous subsection. The biomarker scores were then correlated with MMSE (MiniMental State Examination) as an available measure of AD severity.

## Results

### Biological Age

**Figure 1:**
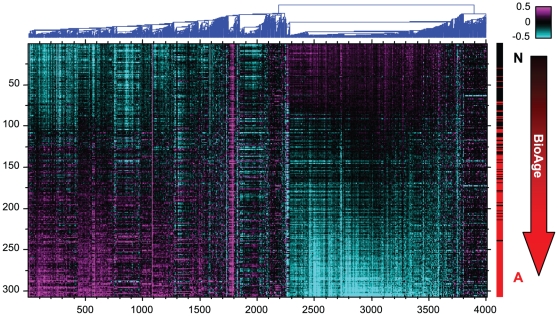
Gene expression in PFC1. The heat map shows hierarchical clustering of the 4000 most variable genes. The samples (rows) are sorted according to the values of the first principal component of the complete dataset and labeled according to diagnosis (normal samples in black, AD samples in red on the right).

Analysis of differential gene expression in prefrontal cortex samples between non-demented individuals and AD patients revealed massive changes, with more than 18,000 transcripts significantly regulated (ANOVA *p*<1E–6) by more than 28% (see Fig. S1). Much of this differential expression was due to a single gene expression pattern that defined the first principal component (PC1) in both AD and normal samples. PC1 explained 45% of variance in the upregulated genes and 60% of variance in the downregulated genes. As shown on the heat map in Figure 1, AD and normal samples dominated the opposite ends of this gene expression pattern, with some subjects from each group in the intermediate range. When normal and AD subjects were considered separately, it was largely the same genes that contributed to the PC1 pattern in both AD and normal samples as shown by correlation analysis in Figure S1. This analysis indicated that the same major biological process reflected in the gene expression started in normal brains and continued developing in AD brains. We found a significant correlation of PC1 with chronological age in non-demented individuals (ρ = 0.58, *p* = 9E–13) and concluded that this gene expression pattern captures normal aging processes in prefrontal cortex. Interestingly, this correlation did not exist in AD patients (ρ = 0.10, *p* = 0.17). Table S2 contains the lists of genes that were most up- and downregulated with age and were selected based on the strongest absolute correlations with PC1.

**Figure 2:**
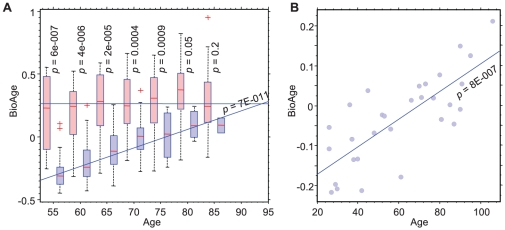
Aging score versus chronological age in PFC1. The box plots (A) demonstrate the distribution of BioAge in different 5-year long age segments and list the ANOVA p-values for the BioAge separation between normal and AD subjects in each chronological age segment. (B) Prediction of chronological age in the independent normal cohort using BioAge. The postmortem prefrontal cortex samples from individual of different age were profiled in an earlier study (GSE1572) [3]. BioAge was calculated based on average expression of several hundred genes from Table S2 (see Methods).

It is useful to ascribe a score based on average expression levels of all included genes as a composite measure (see Methods). We refer to the PC1 biomarker score as BioAge (biological age) based on the hypothesis that the BioAge score for an individual is a more precise and objective measure of the progression of age-related changes than chronological age. Overall, most AD samples attained much larger values of BioAge than normal samples (AUROC = 0.92). See also Table 1 for other characteristics of this biomarker. Comparison of BioAge in AD and non-demented individuals at different chronological age groups revealed a very significant difference at younger ages which decreased in chronologically older age groups. While BioAge of non-demented individuals gradually increased with age, AD patients showed consistently high levels of BioAge regardless of chronological age (Fig. 2A). The extrapolated BioAge of normal subjects would reach average AD levels at the age of 100 years. The most advanced AD brains correspond to an extrapolated age of 140 years in non-demented subjects.

**Table 1:** Biomarker Characteristics.

Biomarker	BioAge	Inflame	NdStress	Alz
Differential variance explained, all samples	0.42	0.23	0.29	0.17
Differential variance explained, BioAge-matched samples	0.09	0.11	0.22	0.06
Coherence in normal samples	0.80	0.80	0.57	0.64
Coherence in AD samples	0.84	0.82	0.76	0.81
AUROC, all samples	0.92	0.88	0.89	0.81
AUROC, BioAge-matched samples	0.69	0.72	0.75	0.69
AUROC, normal and AD samples from PFC2	0.94	0.89	0.87	0.84

The reported values were obtained using PFC1 samples unless otherwise noted.

As an independent test of the power of BioAge to predict normal chronological age, we applied this biomarker to an independent cohort of prefrontal cortex samples from non-demented individuals (GSE1572). This gene expression data were used to qualitatively describe aging in an earlier study [3]. BioAge score in these samples strongly and significantly correlated with chronological age of the subjects in the range from 26 to 106 years (ρ = 0.75, *p* = 8E–7) (Fig. 2B). In addition, BioAge corresponded to the second principal component in the GSE1572 dataset (ρ = 0.90, *p* = 4E–11), validating that aging is a major reproducible source of variance in gene expression in PFC. Similar prediction of chronological age using gene expression was recently proposed [33].

We performed another validation of BioAge as a predictive biomarker of the brain condition in elderly subjects both with and without AD using publicly available gene expression data from hippocampus samples (GSE1297). These data were used in prior research to qualitatively describe AD progression based on gene expression correlation with the disease severity, from control group through severe AD [31], [32]. We found that BioAge score calculated in these samples strongly and significantly correlated with the MiniMental Status Examination (MMSE) (ρ = −0.59, *p* = 4E–4). Figure S2A demonstrates the association between BioAge and AD severity. BioAge corresponded to the first principal component in this data set (ρ = 0.70, *p* = 1E–5), capturing a major source of gene expression variance and an overall brain condition.

The massive gene expression changes associated with aging that we detected involved a constellation of biological processes. A gene set annotation analysis revealed that the genes downregulated with increasing BioAge showed significant enrichment for neuronal and synaptic processes, possibly reflecting neuronal depletion or loss of plasticity (Table S3). The upregulated processes include lipid metabolism, FAK signaling and axon guidance as well as the glial marker, *GFAP* (Table S3). In agreement with an earlier analysis of aging signatures observed in normal brains [2], [3], the upregulated genes contain several oncogenes (*TP53*, *PI3K*, *PTEN*, etc.), shown to be strongly correlated with BioAge in Figure S3.

We also noticed that the genes upregulated with age in normal samples could be further dissected using a metagene discovery approach (see Methods). We focused on the normal samples with relatively low BioAge (BioAge<0) and found a large metagene with exceptionally high mutual correlation between the genes. We named this metagene Lipa because it included *APOE*, *PPARA*, γ-protocadherins, and other genes involved in lipid metabolism, amino acid metabolism and cell adhesion. Other notable Lipa genes included *HES1*, *TGFB2*, *NTRK2*, and *WIF1*. This metagene was much more coherent in normal samples than in AD samples. The corresponding Lipa biomarker indicated an average 3-fold upregulation of these genes early in the aging process. Figure S4 further illustrates the relationships between metagene-based biomarkers and selected component genes mentioned in the text.

### Disease-Specific Biomarkers

**Figure 3:**
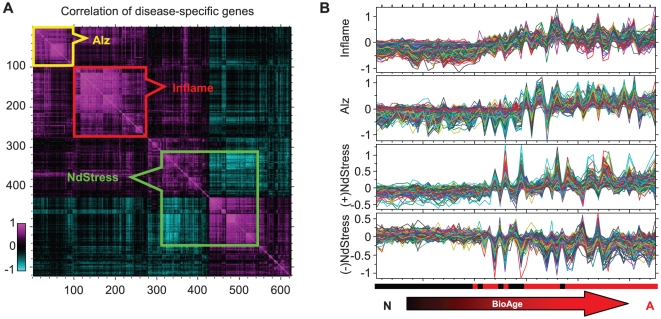
Disease-specific metagenes. (A) Clustered gene-gene correlation matrix demonstrating strong mutual correlations between genes that were differentially expressed between AD and non-demented samples from PFC1. Three outlined clusters correspond to NdStress, Alz, and Inflame. The coregulation of these genes is also shown in the panel (B). Each colored line represents expression levels of individual genes in 55 PFC1 samples from non-demented and AD subjects sorted in the order of increasing BioAge. Only representative samples that scored in the top or bottom 3% for any of the biomarkers were selected for this figure to improve visualization.

Higher BioAge of AD patients explained more than 50% of the differential expression between normal and AD cohorts. In the range of BioAge scores in which AD and normal individuals overlap, there was a significant residual differential expression, composed of several distinct subpatterns that explain a large fraction of the normal-to-AD variance. We focused on 88 AD and 43 normal brain samples with matched moderate levels of BioAge between −0.1 and 0.3. We identified 625 genes that are differentially expressed between the two cohorts (ANOVA *p*<0.005, absolute fold change >25%, FDR<0.1). Figure 3A shows the supervised metagene analysis of these genes based on clustering using gene-gene correlation as a distance measure (see Methods). In this analysis we identified the 3 most regulated metagenes responsible for the majority of the gene expression differences associated with the disease.

**Figure 4:**
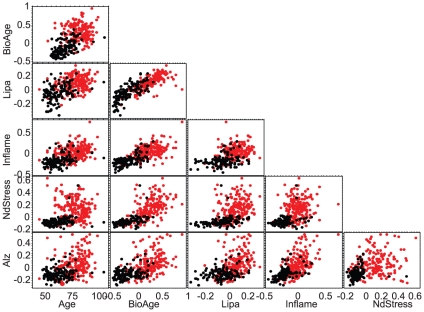
Plot matrix of mutual relationships between key aging and disease-specific biomarkers as well as chronological age. Each biomarker is represented by its score in each sample based on the average gene expression of contributing genes (see Methods). Non-demented PFC1 samples are shown by black dots. AD samples are shown by red dots. All pairwise relationships between the biomarkers and with chronological age are shown.

The first and the largest group of about 2000 genes, further referred to as NdStress, was associated with various metabolic disruptions. This metagene contained some genes that were upregulated and others that were downregulated in AD samples. In normal brain samples with BioAge<0, the expression of these genes was maintained in a relatively stable narrow range with relatively low coherence. In AD samples, however, the expression of these genes varied dramatically and was highly correlated (Fig. 3). Although the plethora of biological pathways reflected in this large metagene precluded significant enrichment of an individual pathway after correcting for multiple testing, the upregulated arm of this metagene contains multiple heatshock and proteosome proteins such as *HSP1A1*, *STIP1*, *HSP1B1*, *PSMB1*/*D6*, and the TGFβ signaling proteins *SMAD2* and *SMAD4*. The downregulated arm of NdStress is enriched in genes involved in folate metabolism, such as *DHFRL1*, *MTR* and *FPGS*, possibly related to the alterations in folate and homocysteine observed in AD patients [34], [35], [36] (Table 2, S2, Fig. S4). Figure 4 includes the relationship between NdStress and BioAge, which moderately correlated in AD samples (ρ = 0.53, *p*<1E–13). At the same time, NdStress and chronological age correlated negatively (ρ = −0.14, *p* = 0.05). This biomarker score explained 22% of variance in differentially expressed genes and demonstrated AUROC of 0.75 in separating AD and normal samples. See Table 1 for other biomarker characteristics.

**Table 2:** Selected pathways that are enriched in metagenes.

Biomarker	Selected enriched pathways
Lipa	Cell adhesion**; RXR function**; fatty acid metabolism**; amino acid metabolism**
(+) BioAge	Molecular mechanisms of cancer*; lipid metabolism*; FAK signaling*; axon guidance*
(−) BioAge	Neuronal activities**, synaptic transmission**; axonal guidance*; long term potentiation/depression**; molecular mechanisms of cancer*; Ca/Glutamate/MAPK signaling*
Inflame	Innate immune response**, apoptosis**, macrophage**
(+) NdStress	Stress response#; PPAR RXR acivation#, glucocorticoid signaling#
(−) NdStress	Metabolic pathways**; folate metabolism#
Alz	Cell communication**; fibrosis**; mesoderm development**; cell adhesion**; ossification*

**Bonferroni corrected Hypergeometric p-value<0.05.

*Bonferroni corrected Hypergeometric p-value<0.1.

# Bonferroni corrected Hypergeometric p-value<0.5.

Up- and down regulated arms of metagenes are denoted as (+) and (−). Complete analysis is shown in Table S3.

The second metagene, further referred to as Alz, consisted of about 200 genes upregulated in AD (Fig. 3). This metagene is enriched in genes involved in cell communication/adhesion, fibrosis, mesoderm development and ossification such as numerous collagen genes, BMP genes, *CTSK*, *MFAP2*/*4*, *FN1*, *VIM*, *WNT6* and *TWIST1* (Table 2, S2, Fig. S4). This metagene also contained several prostaglandin synthases and receptors. Alz positively correlated with both BioAge (ρ = 0.40, *p*<1E–7) and chronological age (ρ = 0.23, *p* = 0.002), see also Figure 4. This biomarker score explained 6% of variance in differentially expressed genes and demonstrated AUROC of 0.69 in separating AD and normal samples (Table 1).

Finally, a small but exceptionally tightly correlated metagene, called Inflame (Fig. 3), contained about 250 genes upregulated with AD, including many inflammation markers, such as *IL1B*, *IL10*, *IL16*, *IL18*, and HLA genes, as well as markers of macrophages, such as *VSIG4*, *SLC11A1*, and apoptosis, such as *CASP1*/*4*, *TNFRSF1B* (p75 death receptor) (Table 2, S2, Fig. S4). Inflame score explained 11% of variance in differentially expressed genes and positively correlated with BioAge (ρ = 0.47, *p* = 1E–10) and chronological age (ρ = 0.28, *p*<0.001) in AD samples. When used as a classifier, the Inflame score was capable of discriminating AD and normal brain with AUROC of 0.69. These genes maintained their mutual correlation in both normal and AD samples but reached significantly higher levels in AD (Table 1).

Figure 4 shows the interplay between the biomarkers discussed above and the complex causal relationships between them. For example, the elevation of Inflame preceded the elevation of NdStress because there are no samples with high NdStress but low Inflame. The correlation between NdStress and Inflame is, however, low in AD samples where NdStress is active (ρ = 0.21, *p* = 0.004). We also observed a low correlation between NdStress and Alz (ρ = 0.21, *p* = 0.004) and moderate correlation between Alz and Inflame (ρ = 0.47, *p* = 1E–11) in AD samples.

We tested performance of these biomarkers in the public gene expression data from hippocampus samples from elder non-demented and AD subjects (GSE1297). We found that at least half of genes in each of the biomarkers preserved their coherence. In addition, we found that Alz was strongly elevated in 5 out of 7 severe cases of AD and correlated with MMSE (ρ = −0.37, *p* = 0.04). Figure S2B shows the distribution of Alz scores among different severity groups. Other biomarkers did not show any significant association with MMSE.

### Systemic and Localized Brain Changes

**Figure 5:**
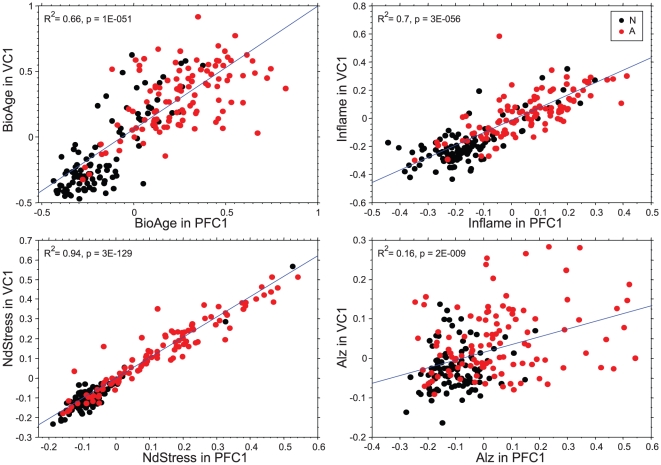
Correlation between biomarker scores in PFC1 and VC1 of the same individuals. Each plot shows relationships between the biomarker values in PFC1 and VC1. Samples from non-demented and AD subjects are shown in black and red respectively.

A unique feature of the HBTRC dataset is the availability of tissue samples from different brain regions from the same individual. All biomarkers in PFC were tested for coherence in visual cortex (VC) and cerebellum (CR). We confirmed that BioAge and the disease-specific biomarkers were also expressed coherently and differentially between normal and AD samples. We then performed direct correlation analysis between the biomarker scores in these regions. BioAge demonstrated relatively high correlations of 0.81 between VC1 and PFC1, with residual differences possibly reflecting different levels of aging between the brain regions. The Lipa biomarker also demonstrated high correlation of 0.80 between these regions. We determined that correlation between Inflame scores in PFC1 and VC1 was equal to 0.83. The highest correlation of 0.93 between PFC1 and VC1 was observed in the NdStress biomarker. These results are also shown in Figure 5, whereas similar observations for PFC1 and CR1 are shown in Figure S5. This exceptionally high level of correlation between the regions is likely explained by the systemic nature of inflammation and metabolic regulation that span diverse brain regions.

**Figure 6:**
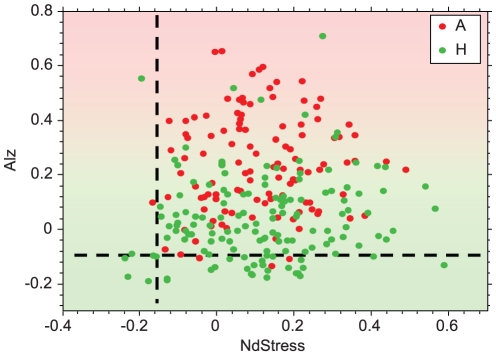
Comparison of NdStress and Alz in AD and HD. AD samples of PFC2 are colored in red. HD samples are colored in green. The reference biomarker scores corresponding to non demented individuals are represented by dashed lines.

Alz scores, on the other hand, did not show any significant correlations between regions in AD samples suggesting that this biomarker is confined to affected brain regions [26] and more specifically related to AD pathogenesis (Fig. 5, S5). Furthermore, the disease biomarkers were fully validated in a hold-out set of samples (Phase 2), which in addition contained some Huntington disease (HD) samples. As shown in Figure S6, BioAge, NdStress, and Inflame were significantly elevated in both AD and HD samples (*p*<1E–6). In general, these biomarkers reached similar average levels in AD and HD samples in all profiled brain regions. In PFC2, however, the average BioAge reached in HD samples was significantly lower than that of AD samples (*p* = 1E–17). These biomarkers, therefore, seemed to capture general systemic neurodegenerative processes rather than being specific to AD. The most striking difference between AD and HD samples was reflected in the Alz biomarker, which again was specific to the presence of AD was not significantly elevated in any brain region in HD samples (Fig. 6).

### Comparison with Brain Transcriptome Modules

Consistent patterns of gene coexpression were recently observed in several large cohorts of brain samples from non-demented individuals [24]. The authors discovered several reproducible modules, which they called brain transcriptome modules, and associated some of them with specific brain cell types. Particularly, modules M4/5, M9, M15, and M16 were associated with microglia, oligodendrocytes, astrocytes, and neurons, respectively. We validated the coherence of these modules in the present HBTRC dataset, and found that more than 90% of genes comprising these modules strongly correlated with each other (ρ>0.7) in non-demented subjects. This analysis supports the finding that the latent structure of gene expression in cortex was preserved in our dataset.

In addition, we compared the gene expression profiling captured by the brain transcriptome modules with BioAge and the other disease-specific biomarkers discovered here. We found a strong correlation between M4/5, associated with microglia, and our Inflame biomarker (ρ = 0.92). In addition, “astrocytic” M15 positively correlates with BioAge (ρ = 0.83) and “neuronal” M16 negatively correlates with it (ρ = −0.93). We also found that none of the major brain transcriptome modules strongly correlated with either the neurodegenerative NdStress or AD-specific Alz biomarkers. This confirms that these expression patterns are novel patterns that can only be detected in brains affected by disease.

## Discussion

### Summary of Molecular Changes in AD

This genome-wide gene expression profiling study of a large cohort of AD and normal aging brains revealed large groups of genes that vary as a function of age and disease status. When the hundreds of gene expression values contained in each of these sets are converted into a single quantitative trait, new molecular biomarkers of biological aging and disease progression emerge. The transcriptional profiles of AD brains were profoundly different from those in non-demented individuals, with thousands of genes differing in their levels of expression between the two cohorts. To reduce the complexity of the observed changes, we focused on key gene expression patterns that explained the most variability across the cohorts. We demonstrated that the most significant pattern in terms of variance explained both within and between AD and non-demented cohorts was BioAge, a biomarker of the level of biological aging in the brain. BioAge captured the extent of gradual molecular changes in the normal aging brain by averaging the gene expression changes associated with a multitude of synchronous physiological events. BioAge can be accurately and reliably assigned to each brain tissue sample in the dataset and used to describe the molecular state of the brain in the same way as we use other clinical and physiological measurements.

Genes upregulated with BioAge point to activation of cell cycle regulation pathways, lipid metabolism and axon guidance pathways (Table S3). Misexpression of cell cycle genes in post-mitotic neurons has been observed in aging and in AD and is postulated to be an important mechanism of neurodegeneration [37], [38]. The enrichment for oncogenes within this set is consistent with biological responses to genotoxic stress activated during aging in an increasingly larger population of brain cells. Genes downregulated with BioAge were associated with a decrease in neuronal activity. Most of these genes maintained a strong correlation (connectivity) with BioAge throughout the entire range of the biomarker. This implies that the core of biological aging is one gradual change rather than several distinct transitions.

Contrary to most aging patterns, a significant loss of connectivity with aging was observed for the Lipa metagene that included *APOE*, *HES1*, and *TGFB2* (Fig. S4). *APOE* and most of the other Lipa genes were expressed at high levels in all AD patients and some normal individuals. This suggests that upregulation of lipid metabolism happens sometime early in the aging process and that activation of *APOE* and changes in lipid metabolism are early precursors of disease possibly related to engagement of protection mechanisms.

We also describe three other distinct disease-specific patterns. The NdStress biomarker, which contained both up- and downregulated genes, dominated differential expression between AD and non-demented brains matched for BioAge score. The upregulated genes contained multiple heatshock and proteasome proteins. Activation of these pathways may reflect the response to disease-related stress. Another set of genes in this module are cell cycle genes indicative of cell cycle arrest or apoptosis. The downregulated arm of NdStress is enriched in one-carbon/folate metabolism genes and could underlay the perturbations in these metabolic pathways, which are among the earliest biomarkers associated with neurodegenerative disorders including AD [34], [35], [36].

The second largest disease-specific pattern (Alz) contained genes associated with cell adhesion, migration, and morphogenesis. This metagene prominently featured genes characteristic of epithelial-to-mesenchymal transition (EMT), such as *VIM*, *TWIST1*, and *FN1* [39], (Fig. S4). The connection of Alz with EMT suggests a major transformation in brain tissue physiology including changes in receptor signaling, growth factor dependence, and cell adhesion during the disease. Considering that the third disease-specific biomarker, Inflame, reflects chronic neuroinflammation [7], [40], it is hard to ignore the similarity between AD with other examples of EMT type 2, such as tissue fibrosis, where chronic inflammation and upregulation of *TGFB2* contribute to pathogenesis [39]. The levels of Alz in AD are much higher than in unaffected brain regions or in the PFC in HD, suggesting that these gene expression changes are not generally reflecting neurodegeneration, but relate to AD pathology.

Finally, BioAge and Inflame are consistent with published analysis of the healthy brain transcriptome and are associated with neuronal, astrocytic, and microglial modules [24]. Importantly, we found that NdStress and Inflame have virtually identical scores in different regions from the same individual. It suggests they measure systemic changes in brain tissue that happen across multiple cell types and layers and are independent of the diverse morphology and makeup of different brain regions. Alz scores, on the other hand, are not the same across all brain regions and had the highest levels in prefrontal cortex, indicating a local rather than systemic nature of EMT.

### Alzheimer Disease Progression Model

Our analysis of gene expression changes in the brains of Alzheimer's patients confirms that AD is both similar and distinct from the process of normal aging. Although each brain was captured only in a particular (postmortem) state and was not studied longitudinally, we can assemble these data as a function of time to propose a few generalized aging trajectories. BioAge and chronological age showed a significant association in non-demented individuals and no association in AD patients, who had consistently high BioAge scores regardless of their chronological age. We attribute this observation to a difference in the strength of the aging drivers, distribution of the aging rates, and different causes of death in the two cohorts. In non-demented individuals, the drivers of aging were weak; the rates of aging were relatively slow and consistent across the population; and, in the absence of unnatural causes, death was likely related to aging issues other than the health of the brain. Since non-demented individuals likely died from causes largely unrelated to neurodegeneration, each individual death is conceptually a random event along the generalized brain aging trajectory. In AD patients, the drivers of aging were stronger and variable across the cohort; and death was generally related to the health of the brain that became incompatible with life regardless of the chronological age. The extrapolated BioAge of normal patients would not reach the highest AD levels until the age of 140 years. Thus, AD can be viewed as an aberrant aging of the brain, which retains the gene expression hallmarks of normal aging combined with additional patterns associated with pathological drivers of the disease and response of the brain tissue to disease-related processes.

For AD patients, we are missing early stages of the aging trajectory and observe only late stages with terminally high BioAge. Unlike the normal cohort that can be represented by a single trajectory, the AD cohort covers a family of trajectories with different rates of biological aging. Patients with a fast rate of biological aging would succumb to disease at younger ages and generally would have higher levels of BioAge relative to their chronological age in the early phases of disease. However, since we do not have longitudinal specimens from subjects before they develop the disease, a second biomarker is required to explain disease progression rates after BioAge is maximal. The expression profile of NdStress fits the properties expected of this progression rate biomarker: it is highest in chronologically young AD patients, and it significantly correlates with (+) BioAge and (−) chronological age. Alz, on the other hand, is the highest in chronologically older patients and does not correlate with BioAge. Thus, patients with high NdStress likely have more accelerated aging trajectories than patients with high Alz. The older chronological age of Alz onset may suggest that the acceleration of BioAge due to Alz does not occur until the level of BioAge of the brain reaches a certain threshold. The quantitative assessment of the brain biological age in terms of BioAge and the rate of its disease-related acceleration in terms of NdStress are two critical hypotheses proposed in this work.

**Figure 7:**
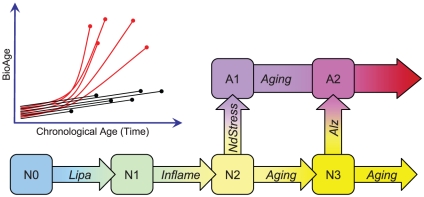
Disease progression model. The trajectories of BioAge changes as a function of time reflect the relatively constant rate of aging in non-demented subjects (black), and acceleration of the rate of aging in AD (red). The dots represent the postmortem state of the brain captured by gene expression profiling. The state transition model defines several broad categories for normal brains N0–N3 and for diseased states A1 and A2. The sequence of transitions and associated gene expression biomarkers are shown by arrows.

Another way to look at the aging trajectory is to model it as a set of molecular transitions that lead to changes in BioAge. Examination of biomarker scores for BioAge-low brains in Figure 4 suggests that upregulation and disruption of Lipa biomarker happens very early in the aging process because most of these samples have the lowest Lipa scores in the cohort. Comparing Inflame with Lipa and BioAge shows that activation of the inflammation biomarker also happens early in the aging process, but not as early as Lipa activation, because there are BioAge-young patients with high Lipa score yet low Inflame. These and other observations can be summarized in the form of a state transition model shown in Figure 7. Aging starts with upregulation of *APOE* and other lipid metabolic genes together with Notch and TGFβ signaling, signifying the transition from N0 to N1. The following upregulation of the Inflame biomarker is associated with transition from N1 to N2. The brains in these states were diagnosed as normal because the subjects did not yet exhibit any cognitive impairment associated with AD. The next transition, from N2 to A1, is associated with massive disruptions in metabolic pathways and marked acceleration of aging follows. Some brains, however, avoid transitioning to A1 and continue to age into N3. Another transition to AD state A2 can happen later, since we observe brains with high scores for both NdStress and Alz, which may be associated with a different path to AD. Alternatively, A2 is possibly localized to a brain region not covered in the dataset. This transition may, therefore, appear later than A1 in a particular brain region and happen much earlier in some other brain region.

The proposed model is most consistent with an age-based hypothesis of Alzheimer's disease that postulates three fundamental steps: initial injury aggravated by age, chronic neuroinflammation, and transition of most brain cells to a new state [8]. These key stages of the disease were independently observed and associated with transcriptional changes in our analysis of the brain transcriptome. We also identified a striking resemblance of the biological processes behind the disease progression biomarkers with epithelial-to-mesenchymal transition (EMT) [39]. The AD processes are most similar to EMT type 2, which is dependent on inflammation-inducing injuries for initiation and continued occurrence. Associated with tissue regeneration and organ fibrosis in kidney, lung, and liver, EMT type 2 generates mesenchymal cells that produce excessive amounts of extracellular matrix (ECM). Similarly, a transition of AD brain into a tissue enriched with mesenchymal cells produces a large amount of ECM containing β-amyloid. This model of the disease implies that multiple independent genetic factors, as well as infections and/or injuries may accelerate consecutive transitions leading to the disease. It also suggests different therapeutic strategies for early and late disease stages. Therapies targeting lipid metabolism and inflammation may be more effective in the early stages. In the late stages, when the brain becomes enriched in mesenchymal-like signaling and adhesion processes, novel approaches that support the survival of the new state of the brain tissue should be considered.

## Supporting Information

Figure S1 **Differential gene expression and variance in PFC1.** (A) Cumulative p-value distribution in the t-test between AD and normal samples. The blue line shows the number of sequences that can be detected for a given p-value cutoff. For example, at p<1E–6, about 18,000 genes can be detected. The green line shows the level of false positives due to multiple testing. (B) Pareto diagram of variance explained by the first 10 principal components. The first principal component dominates the distribution explaining 33% of the data variance. (C) Comparison of correlations between PC1 and individual genes in normal and AD samples. Related to Figure 1.
(PDF)

Figure S2 **Validation of BioAge and Alz biomarkers in GSE1297.** Panel (A) demonstrates the relationships between projected BioAge score and the disease severity as MMSE. The points are colored according to the assigned severity level. The box plots represent the distribution of the biomarker scores in the hippocampus samples from non-demented control subjects and subjects with AD of different severity. Panel (B) shows the same analysis for the Alz biomarker.
(PDF)

Figure S3 **Regulation of selected sell cycle regulation genes with BioAge.** The heat map shows hierarchical clustering of selected 17 genes involved in cell cycle regulation and DNA repair. The samples (rows) are sorted according to the values of the first principal component of the complete dataset and labeled according to diagnosis (normal samples in black, AD samples in red on the right). Related to Figure 2.
(PDF)

Figure S4 **Expression of selected genes and their relationships with biomarkers.** The heat map shows hierarchical clustering of 17 selected genes and 5 biomarkers developed in this work. The samples (rows) are sorted according to the values of the first principal component of the complete dataset and labeled according to diagnosis (normal samples in black, AD samples in red on the right). Only samples with BioAge<0.4 are shown. Related to Figure 3.
(PDF)

Figure S5 **Correlation between biomarker scores between PFC1 and CR1 of the same individuals.** Each plot shows relationships between the biomarker values in PFC1 and CR1. Samples from non-demented and AD subjects are shown in black and red respectively. Related to Figure 5.
(PDF)

Figure S6 **Validation of mutual relationships between key biomarkers in PFC2 cohort, which contained non-demented (black), AD (red), and HD (green) samples.** Compare with Figure 6.
(PDF)

Table S1 **Demographic, clinical, and experimental composition of the HBTRC gene expression dataset.**
(PDF)

Table S2 **Gene sets that comprise gene expression biomarkers of aging and AD progression.**
(XLS)

Table S3 **Functional annotations of the biomarker gene sets based on pathway enrichment. The abridged summary of this analysis is provided in** **Table 2** **.**
(XLS)
