# Supplementary figures and images for: Molecular Insights into the Pathogenesis of Alzheimer's Disease and Its Relationship to Normal Aging
Source: PLoS One. 2011 Dec 28;6(12):e29610. doi: 10.1371/journal.pone.0029610 (PMC3247273; doi:10.1371/journal.pone.0029610)

Figure S1

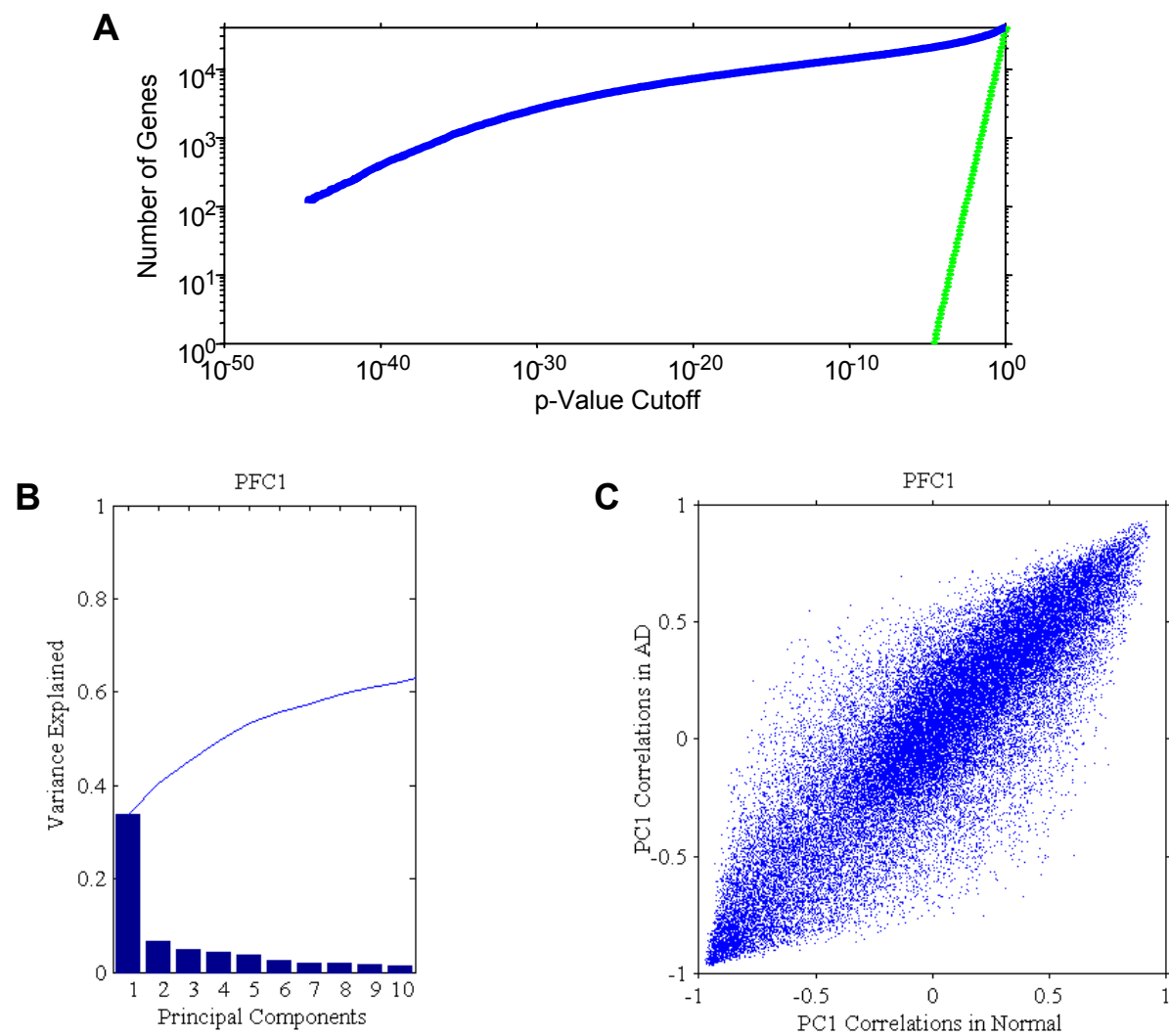

Supplement: Figure S1 — Differential gene expression and variance in PFC1. (A) Cumulative p-value distribution in the t-test between AD and normal samples. The blue line shows the number of sequences that can be detected for a given p-value cutoff. For example, at p<1E–6, about 18,000 genes can be detected. The green line shows the level of false positives due to multiple testing. (B) Pareto diagram of variance explained by the first 10 principal components. The first principal component dominates the distribution explaining 33% of the data variance. (C) Comparison of correlations between PC1 and individual genes in normal and AD samples. Related to Figure 1. (PDF) [file pone.0029610.s001.pdf]

Figure S2

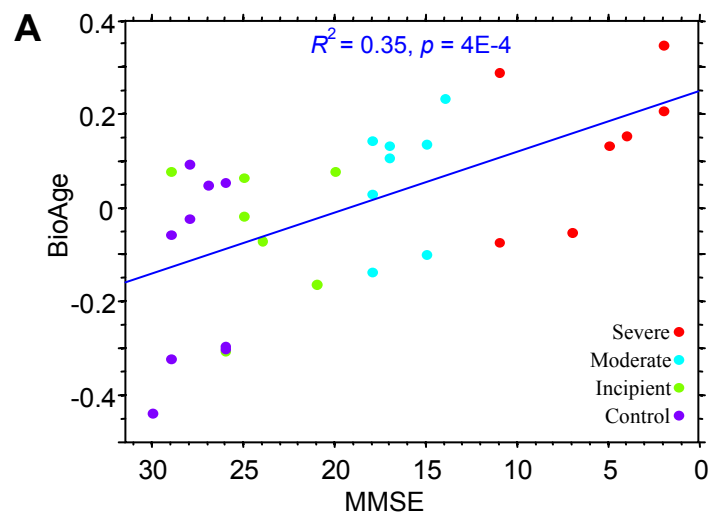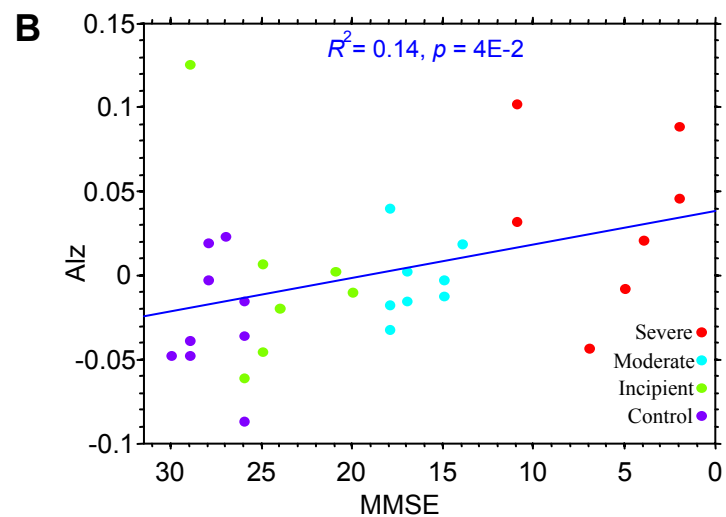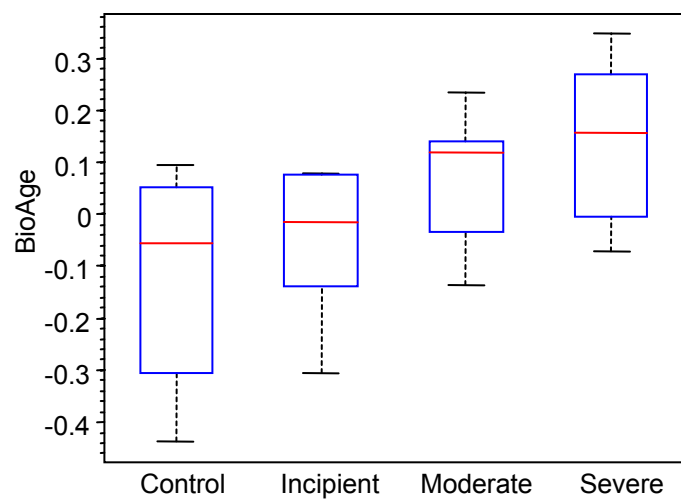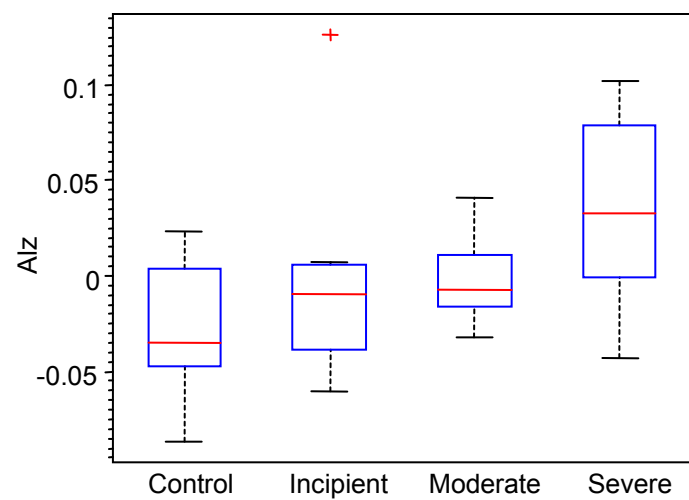

Supplement: Figure S2 — Validation of BioAge and Alz biomarkers in GSE1297. Panel (A) demonstrates the relationships between projected BioAge score and the disease severity as MMSE. The points are colored according to the assigned severity level. The box plots represent the distribution of the biomarker scores in the hippocampus samples from non-demented control subjects and subjects with AD of different severity. Panel (B) shows the same analysis for the Alz biomarker. (PDF) [file pone.0029610.s002.pdf]

Figure S3

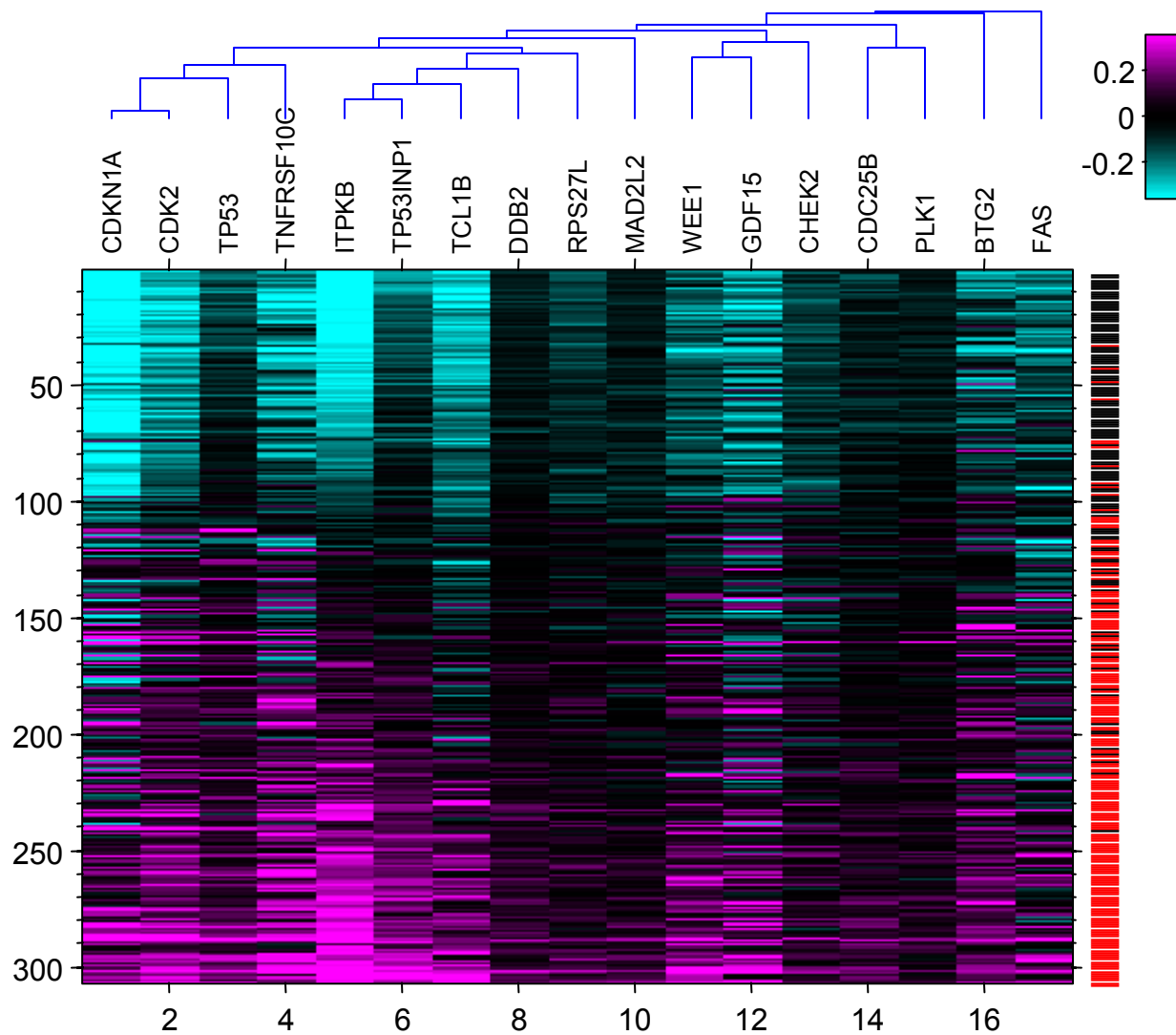

Supplement: Figure S3 — Regulation of selected sell cycle regulation genes with BioAge. The heat map shows hierarchical clustering of selected 17 genes involved in cell cycle regulation and DNA repair. The samples (rows) are sorted according to the values of the first principal component of the complete dataset and labeled according to diagnosis (normal samples in black, AD samples in red on the right). Related to Figure 2. (PDF) [file pone.0029610.s003.pdf]

Figure S4

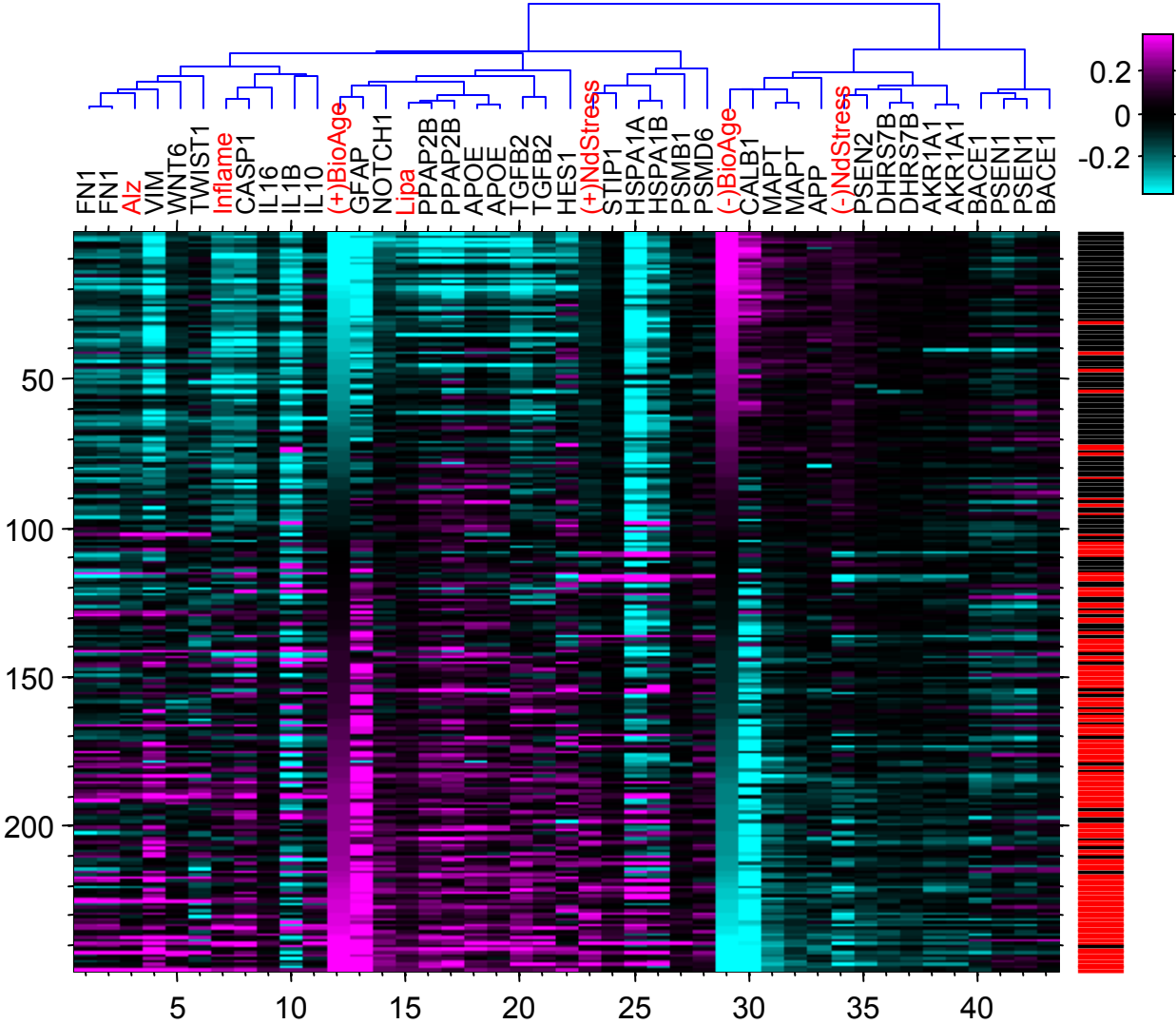

Supplement: Figure S4 — Expression of selected genes and their relationships with biomarkers. The heat map shows hierarchical clustering of 17 selected genes and 5 biomarkers developed in this work. The samples (rows) are sorted according to the values of the first principal component of the complete dataset and labeled according to diagnosis (normal samples in black, AD samples in red on the right). Only samples with BioAge<0.4 are shown. Related to Figure 3. (PDF) [file pone.0029610.s004.pdf]

Figure S5

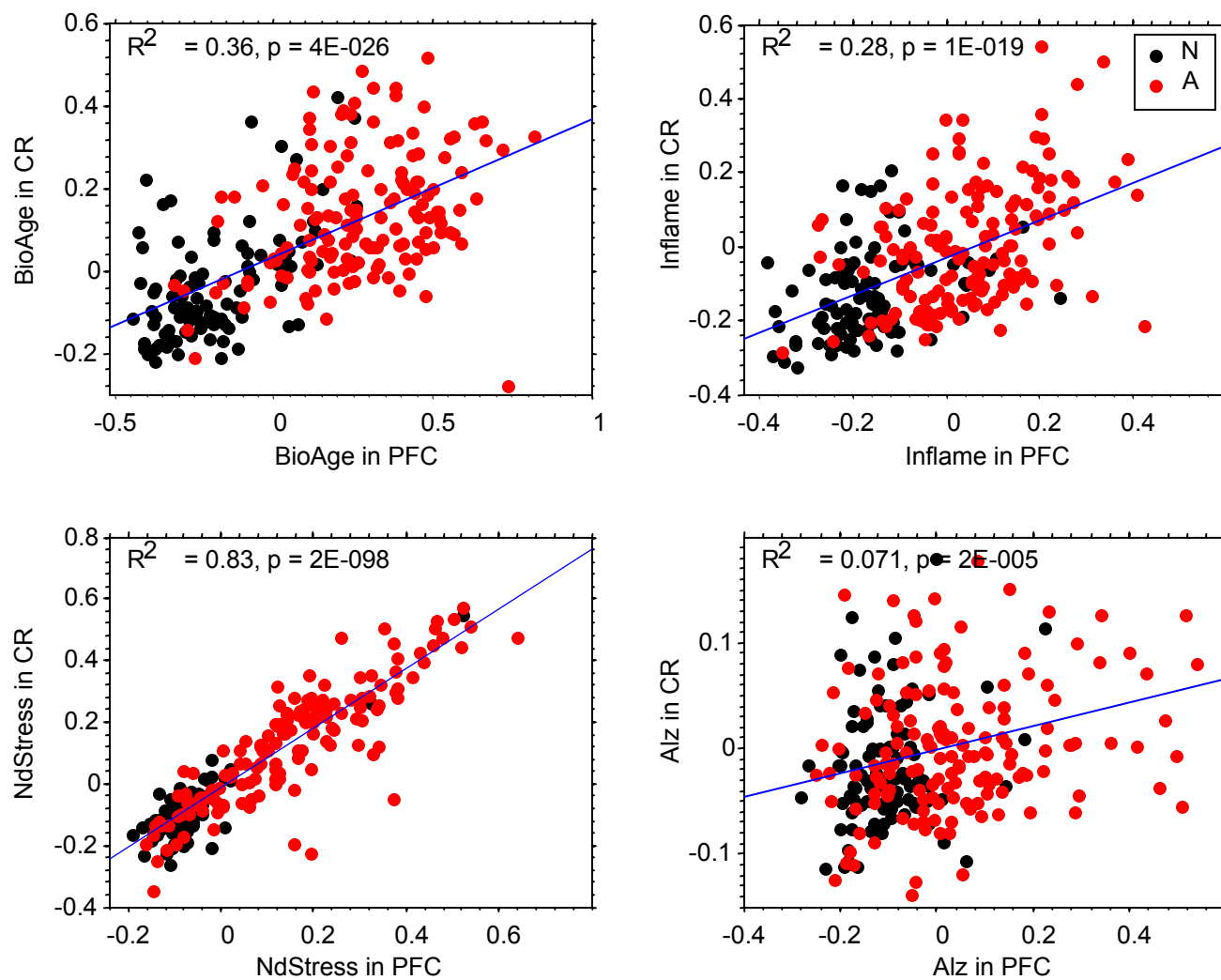

Supplement: Figure S5 — Correlation between biomarker scores between PFC1 and CR1 of the same individuals. Each plot shows relationships between the biomarker values in PFC1 and CR1. Samples from non-demented and AD subjects are shown in black and red respectively. Related to Figure 5. (PDF) [file pone.0029610.s005.pdf]

Figure S6

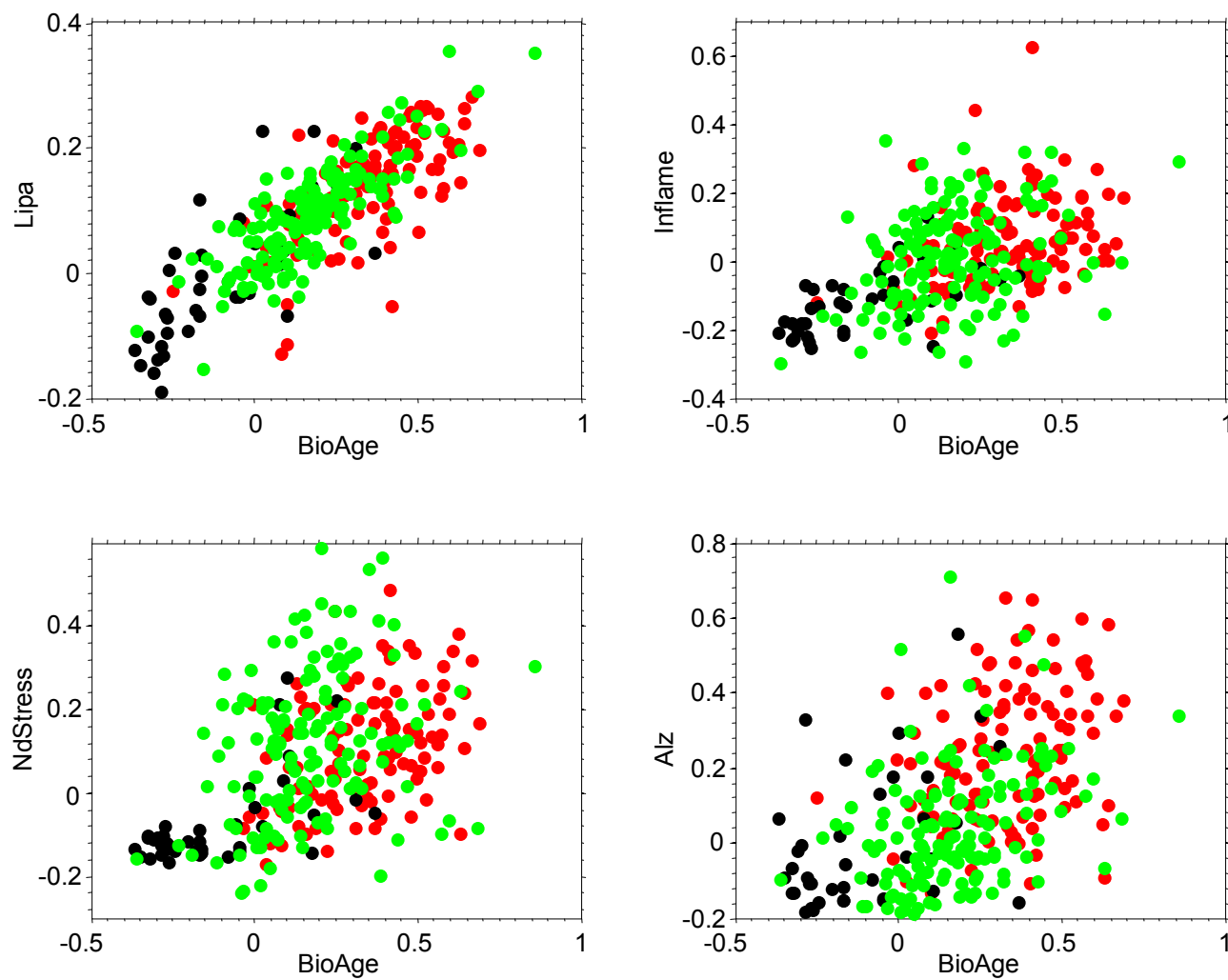

Supplement: Figure S6 — Validation of mutual relationships between key biomarkers in PFC2 cohort, which contained non-demented (black), AD (red), and HD (green) samples. Compare with Figure 6. (PDF) [file pone.0029610.s006.pdf]
