# Supplementary material for: Molecular Insights into the Pathogenesis of Alzheimer's Disease and Its Relationship to Normal Aging
Source: PLoS One. 2011 Dec 28;6(12):e29610. doi: 10.1371/journal.pone.0029610 (PMC3247273; doi:10.1371/journal.pone.0029610)
Supplement: Table S1 — Demographic, clinical, and experimental composition of the HBTRC gene expression dataset. (PDF) [file pone.0029610.s007.pdf]

Table S1

Region, Phase	Diagnosis	Total	Males	Females	Mean Age	Age Range	Mean PMI	Mean Braak Stage	Mean pH	Mean RIN
PFC1	Normal	125	93	32	63.8	22-106	22.2	0.6	6.4	7.2
	Alzheimer	181	81	100	79.7	47-100	14.5	4.9	6.2	6.7
VC1	Normal	104	82	22	63.5	22-106	22.4	1.5	6.4	7.0
	Alzheimer	116	57	59	79.7	47-100	14.1	4.4	6.3	6.7
CR1	Normal	103	80	23	63.3	22-106	22.0	0.5	6.5	6.6
	Alzheimer	173	79	94	79.8	54-100	14.9	4.9	6.4	6.5
PFC2	Normal	38	30	8	63.2	50-86	22.4	0.7	6.6	6.9
	Alzheimer	115	41	74	81.5	59-98	12.6	4.9	6.3	6.8
	Huntington	141	74	67	57.7	21-85	20.8	0.6	6.4	7.3
VC2	Normal	23	18	5	61.0	50-80	22.2	0.8	6.5	7.0
	Alzheimer	53	18	35	81.0	60-95	11.7	5.2	6.2	6.6
	Huntington	132	65	67	56.5	18-93	20.6	0.4	6.4	7.0
CR2	Normal	25	20	5	63.3	50-82	22.0	0.7	6.5	6.5
	Alzheimer	49	17	32	80.1	59-97	13.5	5.0	6.5	6.4
	Huntington	139	72	67	56.3	18-93	20.0	0.4	6.5	6.7
